# Investigation of Plasma-Assisted Functionalization of Graphitic Materials for Epoxy Composites

**DOI:** 10.3390/nano10010078

**Published:** 2019-12-31

**Authors:** Carlo Boaretti, Martina Roso, Renato Bonora, Michele Modesti, Alessandra Lorenzetti

**Affiliations:** Department of Industrial Engineering, University of Padova, Via Marzolo 9, 35131 Padova, Italyalessandra.lorenzetti@unipd.it (A.L.)

**Keywords:** graphite, graphene, functional composites, plasma functionalization

## Abstract

In this study we evaluated the effect of microwave vacuum plasma for the surface functionalization of graphitic fillers (graphite and graphene); we also showed the effect of the functionalization on the mechanical and electrical properties of epoxy composites. Optimized conditions of plasma treatment were defined to obtain high plasma density and increased surface hydrophilicity of the fillers, with high stability of functionalization over time and temperature. However, the extent of such treatments proved to be limited by the high temperatures involved in the curing process of the resin. The use of specific gas mixtures (He/O_2_) during functionalization and the use of a high surface filler (graphene) can partially limit these negative effects thanks to the higher thermal stability of the induced functionalization. As a consequence, mechanical tests on graphene filled epoxies showed limited improvements in flexural properties while electrical resistivity is slightly increased with a shift of the percolation threshold towards higher filler concentration.

## 1. Introduction

Plasma treatment has become a widely employed technique both at laboratory and industrial scale to provide surface functionalization of materials [1]. The process involves the interaction of ions and free radicals on the surface of the sample to be treated and determines a number of different physical phenomena such as ablation [2], etching [3], crosslinking (for polymers) [4], and surface activation [5,6].

An interesting application of this technique is related to the modification of the hydrophobic character of carbon structures in order to improve their dispersion and compatibility with polymeric polar matrices. The final properties of these kinds of composites are mainly dependent on the interactions at the interface between the filler and the matrix. Carbon-based structures like graphite and graphene are capable to produce only secondary Van der Waals interactions, responsible for the pronounced tendency of layers restacking after exfoliation [7] and dispersion in polar media. Without a suitable strategy to improve the chemical affinity with the matrix, this incompatibility leads to the formation of defects and agglomerates capable to alter negatively the properties of the final composites. In order to overcome this proneness to agglomeration, the usual approach is to induce a modification of the surface energy of the filler by covalent or non-covalent functionalization [8,9,10]. Nonetheless, this type of functionalization presents several drawbacks such as the use of aggressive chemicals, the possible introductions of defects in the fillers structure and the use of solvents which reduces the environmental friendliness of the process.

The use of plasma can be a suitable alternative route for the compatibilization of carbon-based fillers with polar media thanks to its low energy consumption, non-aggressive tailored functionalization of surface without modification of bulk properties and up-scalability without the disposal of hazardous materials [11]. Indeed, although predominantly employed for the synthesis of carbon-based materials [12,13,14], lately, plasma technology has been applied for the modification and/or functionalization of graphene [15], showing promising results for applications concerning ultracapacitors [16], electrochemical energy devices [17,18], photocatalysts [19], sensors [20], optoelectronics [21], dye-sensitized solar cells [22], glucose biosensing [23] and DNA detection [24].

Nevertheless, the extent of the modification induced by the plasma treatment depends on several factors [25] such as the specific gas and material to be treated as well as the conditions employed for the treatment. The amount of active species formed during plasma generation depends on factors such as type of discharge for sustaining plasma, discharge power and frequency, gas pressure, volume and shape of the treatment chamber [26]. In turn, the distance between the surface and the probe influences the amount of active species produced which are capable to react on the surface of the material before recombination [25]. Besides that, the combination of plasma gas composition, plasma power and treatment time can influence the qualitative (e.g., type and amount of functional groups and carbon type of hybridization) and quantitative (e.g., generation of defects) modifications of the filler surface [27,28,29,30], with impact on the interfacial interaction with the matrix and consequent alteration of the final properties of the composite. For these reasons an understanding of how the plasma treatment affects the chemical and structural characteristics of carbon fillers and how these modifications alter the properties of final composites is fundamental to understand the suitability of this technique as alternative and competitive solution to traditional industrial practices for composites production.

Plasma functionalization of carbon-based fillers can be beneficial for the development of epoxy composites employed in various high performance applications like adhesives, coatings and structural components for the automotive, aerospace and marine sectors. Such composites could improve the mechanical properties [30] of the base resin in terms of strength, stiffness and toughness, providing lighter components and more durable materials than the actual market benchmarks.

Up to now, many studies have been devoted to the analysis of surface plasma treated fiber-based [31] and carbon nanotubes [32,33,34] based composites but little has been devoted to the study of particulate polymeric composite containing plasma functionalized graphite/graphene.

In their review Alam et al. [35] compared plasma treatment with wet-chemistry for the surface amination of carbon nanoparticles. They observed that although the incorporation of plasma functionalized fillers is beneficial for the mechanical and functional properties of the final composites, the effect of plasma parameters on surface structure and grafting density is still poorly investigated. Zaldivar et al. [30] employed oxygen plasma for the functionalization of graphite nanoplateles dispersed in an epoxy matrix. They observed remarkable mechanical performances and no significant agglomeration, although the comparison was limited to the pristine unfilled resin. Domun et al. [36] investigated the fracture properties of epoxy nanocomposites using low amounts of plasma functionalized graphene nanoplatelets. In their study a significant fracture toughness is achieved without compromising the tensile and thermal properties of the nanocomposites, although with the use of a solvent assisted technique for the better dispersion of the filler.

In this study we have analyzed the effect of different parameters on the optimal plasma-functionalization of graphite/graphene fillers for the production of epoxy composites. The effect of the functionalization was investigated in terms of stability over time and temperature and in terms of impact on the electrical and mechanical properties of the final composites in comparison with their unfilled counterparts.

## 2. Materials and Methods

### 2.1. Materials

The two-components low viscosity epoxy resin, RX771C (bisphenol A diglycidyl ether, DGEBA) and curing agent HX932C (aromatic amine), were supplied by Robnor Resinlab (Swindon, UK) and pre-dispersed at a weight ratio of 100:24. The two fillers employed for the study were CTherm 002 (D_50_ = 39 μm, BET = 25 m^2^/g) expanded graphite, provided by Imerys Graphite & Carbon (CH) (Bodio, Switzerland), and SG221 (D_50_ = 40 μm, BET = 400–600 m^2^/g) chemically reduced graphene oxide, provided by Avanzare S.L. Technological Innovation (ES) (Navarrete, Spain).

### 2.2. Composite Manufacture

A homogeneous dispersion of the filler in the mixture of resin and hardener has been realized with a rotor-stator device (Ultra-Turrax^®^ T25, Ika-Werke, Staufen, Germany) after resin preheating. In case of functionalized fillers, these ones have been dispersed in the mixture immediately after their functionalization. Specifically, the samples were heated in an oven at 80 °C for 15 min, in order to decrease the viscosity and improve their processability, and subsequently homogenized at 7500 rpm for 10 min. The resultant mixture was degassed under vacuum for 30 min and poured into polytetrafluoroethylene (PTFE) molds before curing in oven at 120 °C for 12 h.

### 2.3. Plasma Functionalization

**Figure 1:**
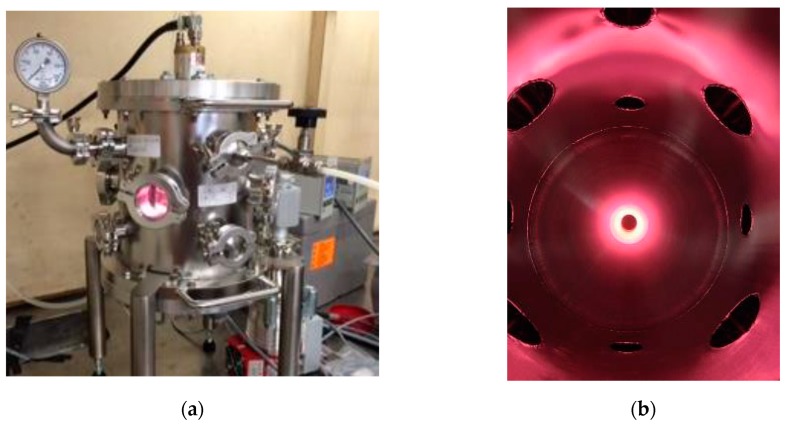
Picture of the chamber for low pressure plasma treatment developed by Sairem ((**a**) external view, (**b**) internal view).

The functionalization of the fillers has been carried out with the use of a custom-made stainless steel reaction chamber (~6l of volume) equipped with and electron cyclotron resonance coaxial plasma source (*Aura-Wave*, SAIREM SAS, Décines-Charpieu, France) as shown in Figure 1. The system was powered by a microwave solid state generator (*MiniFlow 200SS*, SAIREM SAS, Décines-Charpieu, France) capable to provide a robust control over the frequency (from 2.4 to 2.5 GHz with steps of 0.1 MHz) and the level of microwave power (from 0 to 200 W with steps of 1 W).

The low pressure required for plasma generation (10^−4^-a few 10^−2^ mbar pressure range) was achieved by connecting the system to a diaphragm pump (MVP 040-2, Pfeiffer Vacuum, Asslar, Germany) to obtain a primary vacuum and subsequently to a turbopump (HiPace 80, Pfeiffer Vacuum, Asslar, Germany) for high vacuum. Due to the batch nature of the system, suitable for a laboratory scale, every plasma trial has been repeated twice in order to mix the filler between two subsequent treatments and achieve a more homogeneous surface functionalization.

### 2.4. UV-vis Spectroscopy

UV-visible spectroscopy was employed for the evaluation of the sedimentation rate of composite masterbatches before curing using a Lambda 19 UV/VIS/NIR spectrometer (Perkin Elmer, Waltham, MA, USA). It is well known that, under the conditions that satisfy the Lambert-Beer’s law, the absorbance of a solution is directly proportional to the concentration of the solute into the solvent:

(1) $${A=\;\alpha c}_{i}l$$

where *α* is the molar extinction coefficient (dependent on the solvent employed and on the wavelength of the employed ray), *l* is the optical path and *C_i_* is the molar concentration of the sample. These tests were carried out by analyzing specimens of resin containing dispersed filler over time from the same dispersion and evaluating the absorbance at the specific wavelength of 600 nm [37] after dilution with *N*,*N*-dimethylformamide (Sigma-Aldrich, St. Louis, MO, USA) to a dispersion to solvent mass ratio of 1:1. To ensure to consider only the effect due to the sedimentation of the filler, the samples have always been taken from a fixed depth from the free surface of the dispersion before solvent dilution.

### 2.5. Contact Angle Measurements

Contact angle measurements of the powder samples has been carried out with a Krüss Drop Shape Analyzer (KRÜSS, Hamburg, Germany) using ultrapure water to evaluate hydrophilicity. The samples employed were compacted into tablets with the use of a manual hydraulic press before carrying the measurements.

### 2.6. Mechanical Testing

The mechanical tests for the cured samples were carried out using a Galdabini Sun2500 dynamometer (Galdalbini, Cardano al Campo, Italy) with a 25 kN load cell. Mechanical moduli and strengths were calculated according to the ASTM D790 [38] standard for flexural properties and ASTM D638 [39] standard for tensile properties.

### 2.7. Electrical Resistivity Measurements

Electrical resistivity measurements have been carried out for the cured samples at different wt% of filler loadings. These measurements have been realized with an electrometer (model 6517B, Keithley, Cleveland, OH, USA) connected to a guarded text fixture with rounded electrodes (model 8009, Keithley, Cleveland, OH, USA), by applying a voltage of 500 V, using flat circular samples (ASTM D257 [40]); the thickness of each sample was measured and used as input value for the electrometer. The values reported in the following are the mean of 15 measurements, obtained by changing the polarity (±500 V) and by detecting the resistivity values after 60 s of electrification.

### 2.8. Dynamic-Mechanical Analysis (DMA)

DMA tests were carried out with Q800 (TA instruments, New Castle, DE, USA) analyzer. The samples were tested with a single cantilever clamp with a fixed strain of 50 μm applied at 1 Hz of frequency over the temperature range between 25 and 200 °C, at a fixed heating rate of 5 °C/min. The glass transition temperature (T_g_) was identified by the peak in the tanδ curve.

### 2.9. Environmental Scanning Electron Microscope (ESEM)

Morphology of the liquid-nitrogen brittle fracture surface of the filled samples has been analyzed by means of Environmental Scanning Electron Microscope (ESEM), (Obdu-cat CamScan MX2500; Cambridge, UK).

## 3. Results

### 3.1. Plasma Functionalization

The first part of the study has been devoted to the screening of the operative conditions for the optimal plasma functionalization of the graphitic fillers. Due to the nature of the equipment employed, the variables taken into considerations are those reported in Table 1. The evaluation of effect of the different levels or conditions of these variables was carried out by checking the extent of wettability of the resulting powders by contact angle measurements with ultrapure water. Indeed, it has already been proved that surface oxygen functionalities provide polar centers to attract water molecules and enhance the surface hydrophilicity, thus lowering the water contact angle (WCA) values while surface C−H bonds contribute to the carbon hydrophobicity and higher WCA values [41]. He et al. [42] showed that a correlation existed between the intensity of the C–O/OH component of the C 1s peak in X-ray Photoelectron Spectroscopy (XPS) analysis of graphitic filler and observed water contact angle; similar conclusions have been reached by Lin et al. [43]. Based on this, the potential impact of the plasma treatment can be easily assessed by contact angle (CA) measurements providing a simple and time-effective tool to evaluate the presence of functional groups containing oxygen on the filler surface. The use contact angle CA measurements for plasma-treated nanomaterials, such as CNTs and graphene, have already been used to assess the functionalization of graphitic fillers [5]. It has also been already been proved that graphitic fillers, which are normally hydrophobic materials, if made hydrophilic, can be incorporated and distributed homogenously within composites to maximize the structural capabilities of the reinforcement [5,44].

A first series of experiments has been carried out by using graphite in order to define optimal conditions for the plasma functionalization to be applied for the case of graphene. Due to the high amount of variables involved, both categorical and numerical ones, and their different selected levels, it has been necessary to realize a subset of all the possible experiments in order to obtain a screening of the most relevant factors. The first approach has been to fix the amount of treated material to lowest value and analyze the effect of the different operative variables. The results, presented in Table 2, have been interpreted by comparison with untreated graphite, which has shown a water contact angle of 65.4 ± 2.3°. The measurements clearly show that a key parameter for an effective graphite plasma functionalization is the gas pressure within the chamber. The best results in terms of wettability are obtained with a higher pressure (0.06 mbar) than that (0.01 mbar) required to maximize the plasma efficiency (minimization of the reflected power). A second important parameter has been identified in the distance between the material to be treated and the plasma source. The lower distance employed (3 cm) has demonstrated to provide better results in reducing the water contact angle, providing complete wettability in conjunction with the higher pressure, quite independently on the microwave power employed.

These results are related to the variation of plasma density that increases in a roughly linear proportion by decreasing the distance of the material from the source and by increasing the gas pressure inside the chamber. The higher the plasma density (percentage of ionized gas particles), the higher the density of the reactive species in the reactor and more pronounced the effect and the stability of the functionalization over time. The optimal conditions identified requires the functionalization of 0.25 g of material for 2 min in presence of 0.06 mbar of oxygen atmosphere and a plasma power of 80 W with a plasma source to sample distance of 3 cm. Once defined the optimal values for these parameters, the other operative conditions seem to have little effect on the wettability of the material. In particular, it can be noted that increasing the time of the treatment (5, 10 min) and the power (100, 200 W) does not provide significant variation of the results, which is a good indication in terms of time and money savings. On the other hand, between the gases employed for the treatment, oxygen seems to provide more significant hydrophilicity, probably because of the generation of different types of oxygen rich functional groups [28,45]. By increasing the amount of material to be treated (from 1 to 5 g), with the same optimal conditions, a good functionalization is still obtained but with less pronounced hydrophilicity and stability over time. In this case a good wettability can be retained for at least 1 month contrary to 75 days as for the samples treated with a smaller amount of filler (0.25 g).

In the case of graphene (initial contact angle of 109 ± 1.5°) suitable operative conditions seem to be similar to those employed for graphite, with consistent functionalization over time even with the employment of only air instead of pure oxygen, as shown in Table 3. This can be ascribed to the higher aspect ratio of the filler, which favors the interaction of the ionized gas molecules over the graphene surface, and the lower amount of materials that can be treated (0.05 g) due to its lower apparent density with respect to graphite. However, the latter is not a concerning limitation since lower amounts of graphene are generally required in the final resin to obtain improvements in both electrical and mechanical properties as well as to maintain adequate viscosity for achieving a better dispersion. The higher degree of wettability achievable with graphene has allowed to extend the range of gases to be employed for the functionalization by taking into consideration also noble gases (He, Ar) and their mixtures with oxygen as can be seen from Table 4. In particular the attention was focused on the He/O_2_ mixture for its capability to provide high energy on the surface of the particles with short treatment times [46], using different volumetric ratios between the two gases to carry out the functionalization tests. This choice was also supported by the higher efficiency of the plasma treatment in terms of lower reflective power obtained with respect to the employment of simple O_2_ or Ar/O_2_ gas mixture.

### 3.2. Stability Tests

**Figure 2:**
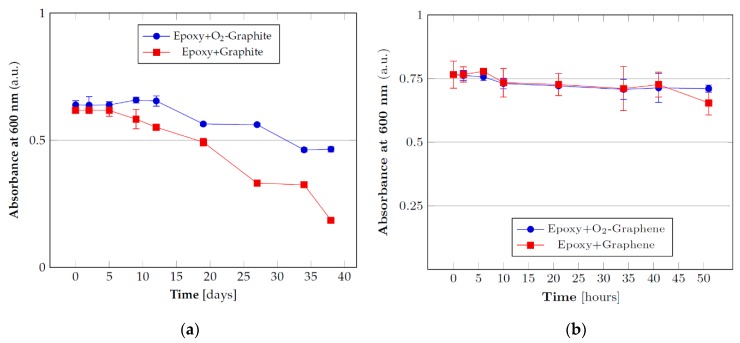
UV-visible absorbance of the diluted resin systems with pristine and functionalized fillers for the evaluation of the dispersions stability: (**a**) 2% graphite and (**b**) 0.05% graphene dispersions.

Once identified the best parameters in order to ensure a suitable and durable functionalization, subsequent tests have been conducted in order to evaluate the stability of the resin masterbatches containing functionalized fillers, a critical issue for particulate composites. In other words, the effect of functionalization on filler sedimentation during both masterbatches storage and resin curing have been investigated by UV-visible spectroscopy. In this manner, it is possible to observe the decrease of filler concentration in the resin by measuring the decrease of absorbance over time as a consequence of the sedimentation process. The tests have been carried out with pristine and functionalized graphite/graphene using reference concentrations between those employed for the subsequent tests (specifically at 2% for graphite and 0.05% for graphene). The tests carried out with the graphite in pure resin (Figure 2a) have shown that with plasma functionalization there is a significant difference in stability starting after 5 days or more of storage, which can be good to guarantee extended storage time for the material. This suggests that the functionalization is capable to provide a more pronounced interaction between the filler and the resin, retarding the sedimentation due to the gravity force.

In the case of graphene (0.05% of filler in resin) the trend for pristine and functionalized samples are almost constant over time and close to the starting value (Figure 2b). This trend can be explained in terms of the high viscosity of the dispersion due to the high surface area of graphene which determines an increased surface contact area with the resin and thus a higher friction that considerably slows the sedimentation process. This result shows that it is possible to store pre-dispersed resin/graphene systems even for a prolonged amount of time without significant variation in the homogeneity of the dispersion. However, in order to better appreciate the effect of the material functionalization by plasma treatment on sedimentation during the curing process, several diluted masterbatches have also been analyzed. Indeed, during the curing process, there is an initial decrease in the resin viscosity due to heating before the following viscosity increase, due to crosslinking process. Such initial reduction in viscosity has been simulated by dilution of the masterbatches with solvent. To this aim, the samples were diluted in *N*,*N*-dimethylformamide, reaching a final concentration of 1% and 0.03% for graphite and graphene, respectively.

**Figure 3:**
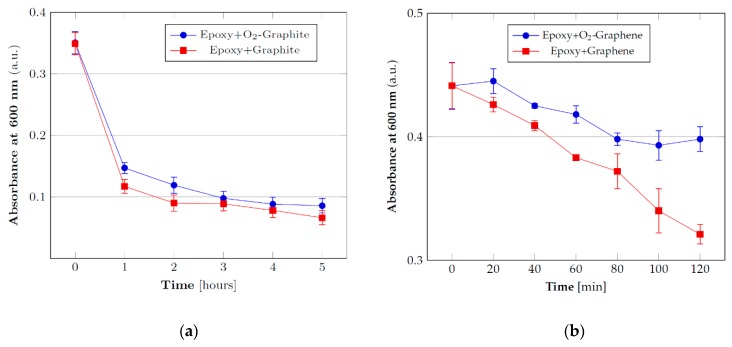
UV-visible absorbance of the diluted resin systems with pristine and functionalized fillers for the evaluation of the dispersions stability: (**a**) 1% graphite and (**b**) 0.03% graphene.

The results reported in Figure 3a show that in the case of graphite a difference in the sedimentation rate for pristine and functionalized filler is still present although less marked than the previous situation without dilution. The effect of the functionalization on the graphene sedimentation in the diluted resin samples is provided in Figure 3b. As in the case of graphite, the filler tends to sediment more quickly but the functionalization provides more stable dispersions thanks to the better interaction with the polar matrix.

These tests, which can simulate the conditions at the beginning of the curing reaction when it is more likely to have agglomeration phenomena, have shown that, even under conditions of low viscosity, the functionalization provides better results than the pristine filler, mainly in case of graphene.

### 3.3. Electrical Resistivity Measurements

An aspect of interest for the practical applications of particulate composites containing electrical conductive fillers is their volume resistivity. Epoxy resins are excellent electrical insulating materials that normally found applications in coatings and as encapsulation material for electrical and electronic components. The possibility to impart electrical conductivity to these materials combined with their excellent mechanical properties and thermo-chemical resistance potentially opens to the opportunity of their employment in various engineering applications such as antistatic coatings, electrical conductive adhesives, electromagnetic interference shielding materials for electronic devices, thermal interface materials, etc. However, although the functionalization of carbon-based filler represents a potential solution to improve mechanical properties by better compatibility with the embedding epoxy matrix, the effect on the electrical conductivity of the resulting composites is still not clear due to different evidences emerging from studies available in literature [47,48,49]. These differences could be ascribed to several factors among which the type of functionalization could represent an important aspect to take into consideration in terms of better dispersion and surface defectivity. For the present case, the relevance of such analysis is related to the absence of data regarding the effect filler functionalization produced by plasma treatment.

**Figure 4:**
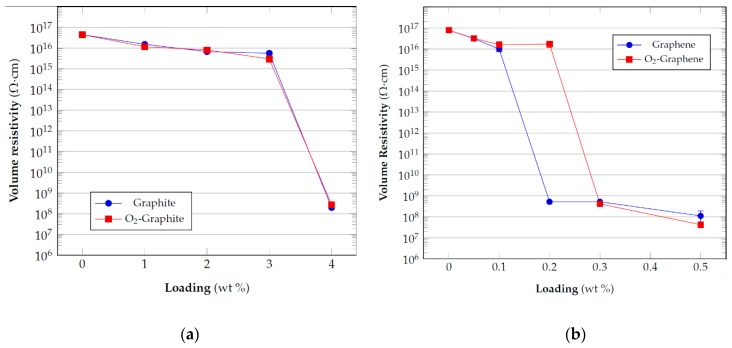
Electrical resistivity of epoxy composites with: (**a**) graphite and (**b**) graphene.

The results of the measurements of volume resistivity realized, reported in Figure 4, have shown that, at least for the concentration values taken into consideration for these tests, there is a similar behavior between the two types (pristine and functionalized) of graphite, with a percolation threshold between 3 and 4% by weight of the filler. In the case of graphene, the trend shown by the plasma treated samples is different from that of the pristine material. The latter already percolates at 0.2 wt% of filler loading while the oxygen functionalized material only percolates at 0.3 wt%.

It has already reported elsewhere [50,51] that oxygen plasma treated carbon materials generally increase their electrical resistivity and the results of the electrical resistivity can be understood considering that the presence of oxygen functionalities determines an increase in the disorder and defectivity of the material structure due to the disruption of the sp^2^ graphene lattice. In this manner, the oxygen functionalities act as scattering sites and negatively influence the electrical transport properties of the treated materials.

The difference in the results between graphite and graphene can be associated to the different extent of functionalization. Yu et al. [50] observed that the increase in electrical resistivity of carbon nanotubes (CNTs) yarn and sheet is proportional to the extent of oxidation induced on the material by the plasma treatment. As a consequence, the lower amount of material treated for graphene (i.e., high plasma power density applied) can determine a higher amount of functional group on its surface and thus a higher resistivity of the filler with respect to the pristine graphene, which has a very low resistivity. For graphite there is probably a lower extent of functionalization which determined a similar electrical behavior to the pristine reference at least for the percentages tested.

### 3.4. First Mechanical Results

**Figure 5:**
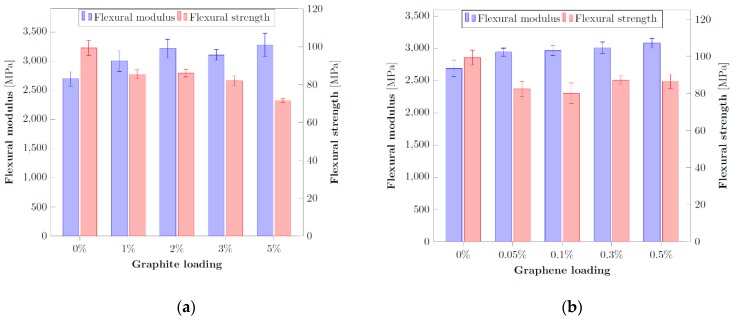
Comparison of the flexural properties for different level of pristine filler loadings: (**a**) graphite, (**b**) graphene.

The effect of the functionalization induced on the graphitic materials by vacuum plasma has been evaluated also by mechanical testing of the flexural properties of the resultant composites with epoxy matrix. Flexural properties of epoxy composites can experience a significant increase upon the addition of graphitic fillers but the extent of this improvement is dependent on several factors like filler concentration, particle size, material processing, extent of filler agglomeration and interfacial bond between the filler and the matrix. Among them, in many studies, the agglomeration is considered the main issue to explain the fluctuant behavior of such property upon filler addition. If the filler is not properly dispersed, the aggregates form stress concentration centers which determine a decrease in the material properties [52]. Such aspect influences both the modulus and the strength of the composite since a bad dispersion is usually accompanied by a weak interface between the filler and the matrix. It is, indeed, such weak interface that prevents a correct transfer of load from the matrix to the filler, which acts as stress concentrator [53]. On the contrary a good exfoliation and dispersion of the filler increases the extent of the interaction area with the matrix. However, the extent of this interaction is strictly dependent on the compatibility between the two. A higher compatibility generates a positive interaction capable to promote a strong filler/surface interface without the presence of defects or voids. In this manner stress transfer efficiency from the matrix to the stiffer filler is promoted and the final composite is capable to acquire higher mechanical performances. For this reason, filler functionalization is a common solution capable to reduce the agglomeration between the particles and increase the interfacial bond with the matrix, determining better mechanical properties with respect to the pristine filler dispersed in the resin at the same level of loading. From this point of view, firstly, initial screening tests on the untreated fillers have been realized in order to define their proper amount to obtain the best improvements for flexural properties and subsequently used as reference for the comparison with the functionalized counterparts. Figure 5a,b show the properties of the composites with the two different pristine fillers (graphite and graphene) in terms of flexural modulus and strength. Graphene amounts tested are extremely lower than those of graphite since it is already well known that very low amounts of graphene are generally required in the final resin to obtain improvements in both electrical and mechanical properties.

As can be seen from the results, there is not an optimal concentration of the filler in the matrix capable to provide the best improvements for all the flexural properties of the final composite. For both graphite and graphene, the addition of a higher amount of filler increases the stiffness of the material (i.e., modulus) but without a concurrent improvement in strength, as a consequence of the poor interfacial interaction with the matrix [54]. This opposite effect is a result already outlined in other studies [48,55,56] and can be ascribed to the previous description of the role of matrix-filler compatibility. Due to the nature of the carbon-based fillers, which are not polar compounds, there is not a good compatibility with the epoxy matrix. This condition determines a higher probability of filler particles agglomeration with the concurrent generation of stress concentrators. As a consequence there is a premature failure of the composite even in presence of a stiffening effect, due to the rigid nature of the filler, in the elastic region of the mechanical behavior.

In the case of graphite, a trade-off value of 2 wt% to obtain good improvements in the modulus without sacrificing the ultimate strength seems to be adequate. On the other side, for graphene composites there is, more or less, the same decrease of strength for all the fillers levels analyzed. For this reason, and to keep the final cost of the composite as low as possible, further investigations have been carried out on 0.05 wt% of graphene.

**Figure 6:**
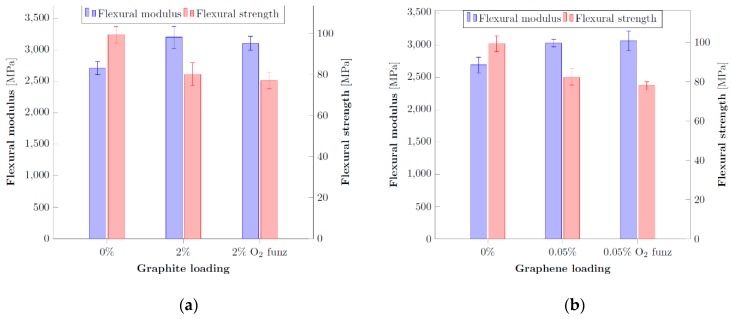
Comparison of the flexural properties for different level of optimal loadings with or without filler functionalization: (**a**) graphite, (**b**) graphene.

Once defined these optimal concentration (i.e., 2 wt% for graphite, 0.05 wt% for graphene), their properties have been compared with the same amount of oxygen-functionalized fillers obtained with the optimal plasma parameters previously identified. From the comparison shown in Figure 6 it is possible to observe that, although improved with respect to the neat resin, there is a negligible difference between the average values of the flexural modulus between the functionalized and non-functionalized samples for both graphite and graphene. On the other hand, the flexural strength is reduced to a slightly lower value with respect to the samples containing untreated fillers as if no better interaction has been determined between the filler and the matrix in presence of surface rich functional groups. A possible explanation of the poor mechanical performances obtained is a potential loss of the functionalization effect on the fillers due to the high temperature (120 °C) necessary to obtain the crosslinking reaction. To validate this hypothesis, it is reasonable to investigate if the plasma functionalization is thermally stable.

### 3.5. Effect of Thermal Treatment on Graphene Plasma Functionalization

To investigate the stability over time of the plasma functionalization at high temperatures, the variation of wettability, before and after the thermal treatments of functionalized graphene, has been measured. This filler has been taken as a reference for the subsequent analysis because its higher surface area with respect to graphite is beneficial in achieving a higher degree of functionalization. For this analysis, different temperatures and treatment times have been taken into consideration in order to understand the stability of the functionalization to such conditions. It is indeed important to remember two important aspects:

- The dispersion (resin + filler) is subjected to heating up to 80 °C for a reduced amount of time (about 1 h) both during the preparation of the samples before the dispersion by homogenization and before the curing starts, during the vacuum treatment to remove the presence of bubbles in the dispersion;
- The curing process is carried out at high temperatures (120 °C) for 12 h but the main concern is related to the first hours of the process where the high temperature lowers the viscosity of the dispersion and agglomeration/sedimentation can take place. After this time the extent of the curing reaction is such to significantly increase the viscosity and “freeze” the particles of the fillers inside the matrix, preventing sedimentation and aggregation.

The results reported in Table 5 show that heating the plasma functionalized material decreases the wettability and determines a loss in the extent of the functionalization, although with different results according to the type of gas employed for the functionalization.

By carrying out the functionalization in presence of oxygen already after 2 h at 80 °C the material loses most of its functionalization, with a similar trends in presence of pure noble gases. The best results can be obtained using a He/O_2_ mixture with a 5:1 volumetric ratio for which the functionalization is stable after a thermal treatment for 1 h at 120 °C and there is only a partial loss of wettability after 12 h at the same temperature.

### 3.6. Optimized Composites Properties

**Figure 7:**
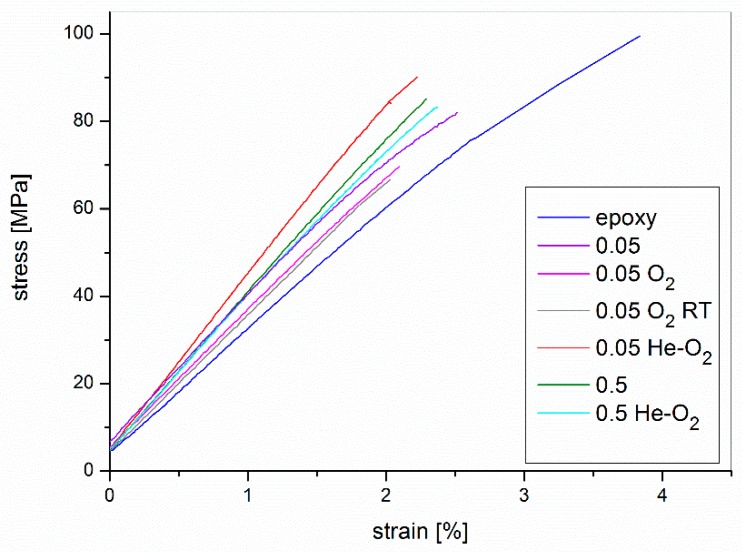
Stress-strain results of flexural tests for pristine epoxy and epoxy/graphene composites treated by plasma functionalization (RT = room temperature).

Since the tests at high temperature have demonstrated how the choice of a suitable gas for the plasma treatment is essential to retain the acquired functionalization during the curing process, a series of composites has been realized and their mechanical and electrical properties have been evaluated on the basis of the previous results. He/O_2_ mixture (volume ratio 5:1) was selected as functionalization gas to compare the results with the pure resin, the composite with pristine and oxygen functionalized filler. Two different percentages were taken into consideration (0.05 and 0.5%) to compare the results for low (where the pristine filler showed better mechanical performances) and high (where there is electrical percolation) filler loadings. A further comparison has been also realized by carrying out the crosslinking reaction at room temperature for seven days (followed by a post-curing step at 120 °C for 8 h) in order to evaluate if the loss of functionalization, with oxygen treatment, takes place also at room temperature; for this composite the dispersion has been carried out in usual conditions (1 h at 80 °C). The stress-strain curves of flexural tests are reported in Figure 7 along with toughness values (Table 6). It can be noted that all filled samples as well as neat epoxy showed brittle behavior, as expected. The toughness of the composites was lower than that of unfilled resin. This implies that the dispersion method used here, although it was not the main subject of the paper, is not optimized [36]. It can also be noted that 0.05% O_2_ and 0.05% O_2_ RT showed the lowest toughness, lower also than that of 0.05% unfunctionalized filled composite. This could be explained considering that the O_2_ functionalized fillers lost their functionalization, as shown before, but maintained the defects due to plasma treatment. The presence of the defects reduced the load transfer efficiency in graphene sheets, and brought about serious local stress concentrations in regions surrounding the defects. It is believed that bigger size defect would result in stress concentration of composites in a larger area. According to brittle failure mechanism, the fracture stress is inversely related to the length of defects [57]. The 0.05% He–O_2_ sample showed the highest toughness among filled samples, owing probably to a better dispersion of the graphene in the matrix with respect the other samples because of the functionalization of the fillers. The toughness of the 0.5% composites (both pristine and functionalized) was lower than 0.05% He–O_2_ one, due to the presence of larger agglomerates which represented stress concentrators. These results are in agreement with SEM observation reported below.

**Figure 8:**
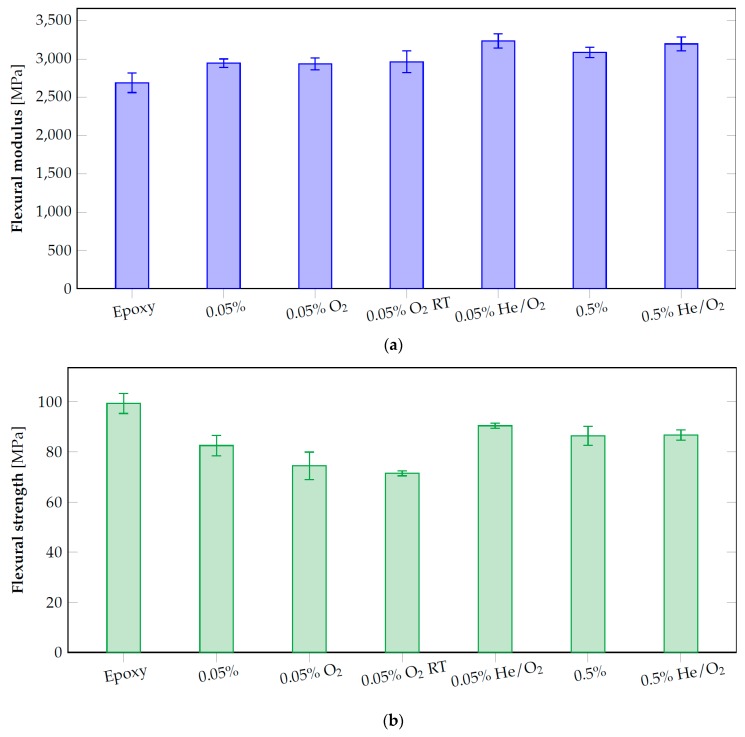
Flexural modulus (**a**) and flexural strength (**b**) comparison for pristine epoxy and epoxy/graphene composites treated by plasma functionalization (RT = room temperature).

Flexural modulus and strength are presented in Figure 8, for comparison.

Considering flexural modulus and flexural strength, the higher improvement was obtained for the samples filled with 0.05% of graphene treated with the He/O_2_ mixture which had better performances than pristine and oxygen plasma treated graphene. The samples treated by oxygen plasma and cured at room temperature did not show improved results with respect to the thermal treated counterpart. As a consequence it is clear that also the preparing steps for the filler dispersion influence the plasma functionalization with oxygen which is not stable even at 80 °C and for small amount of time.

**Figure 9:**
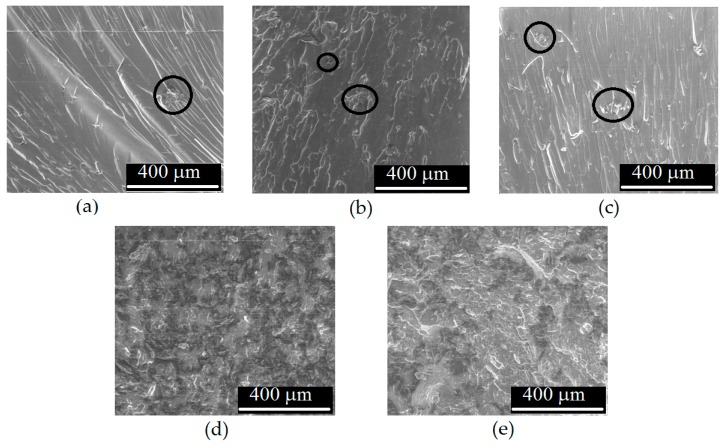
SEM images of brittle fracture surface of (**a**) 0.05%, (**b**) 0.05% O_2_, (**c**) 0.05% He–O_2_, (**d**) 0.5% and (**e**) 0.5% He–O_2_ graphene/epoxy nanocomposites (in (**a**), (**b**) and (**c**) black circles show graphene particles while in (**d**) and (**e**) graphene particles are the darker zones).

Analyzing SEM images of the brittle fracture surface of the nanocomposites (Figure 9), it can be seen that around the graphene particles in pristine and O_2_-functionalized 0.05% graphene samples there were some voids (see black circle in the figure), i.e., there was not good adhesion between filler and epoxy matrix, while in the 0.05% He–O_2_ functionalized sample the filler and the matrix were in more intimate contact, thus leading to a better stress transfer from matrix to filler. For pristine 0.5% graphene samples, the SEM image (Figure 9d) showed very large aggregates while for 0.5% He–O_2_ sample (Figure 9e) the dispersion was better but there were still several large aggregates which acted as stress concentrators. This can explained why in the mechanical tests the increase from 0.05 to 0.5% of graphene did not lead to any appreciable improvement of mechanical properties and the toughness was lower for higher graphene content.

**Figure 10:**
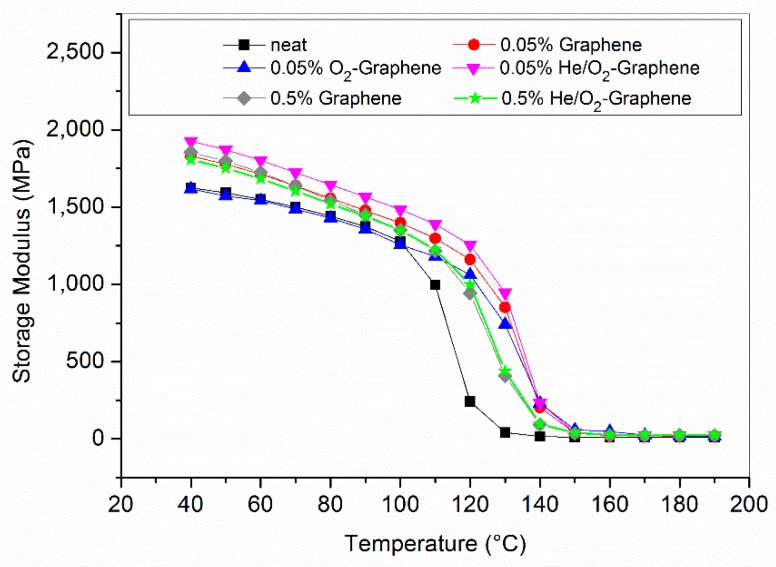
Storage modulus curves for neat resin, 0.05 and 0.5% graphene filled resin.

**Figure 11:**
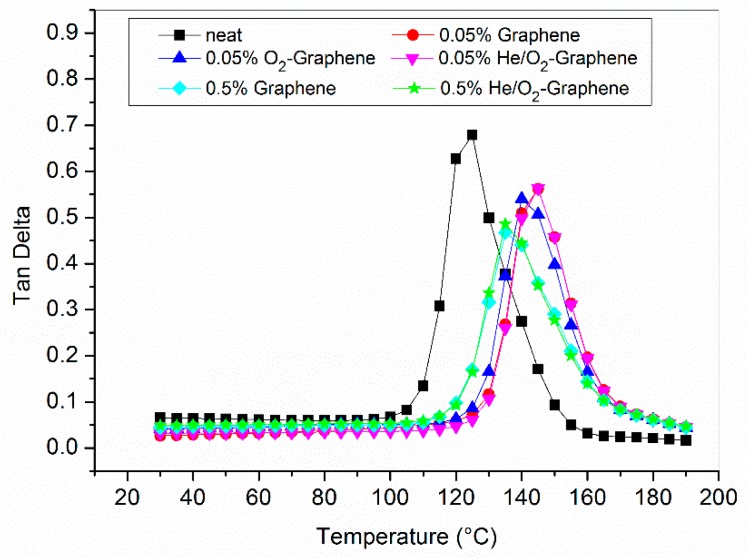
Tan δ curves for neat resin, 0.05 and 0.5% graphene filled resin.

Optimized graphene filled epoxy composites have also been analyzed by DMA and the results are shown in Table 7 and Figure 10 and Figure 11. As can be seen, the introduction of graphene in the matrix is generally followed by an increase of 10–20 °C of the glass transition temperature with respect to the neat resin. This is generally interpreted as a consequence of the fact that the presence of the filler restricts the polymeric chain mobility which necessitates of higher thermal energy to achieve the glass transition [47,58,59].

In this case the functionalization of the filler does not provide any relevant contribution on the thermal property of the material while a lower increase in the value of the T_g_ seems to be associated with the use of the higher concentration of graphene between those investigated. This can be partially explained in terms of the higher difficulty in achieving a good and uniform dispersion, even with an increased compatibility between filler and matrix, in presence of such a high concentration of graphene in the epoxy resin [60]. As a consequence, there is a higher probability to have a re-agglomeration of the dispersed particles during curing, with a reduction in the total interfacial surface between the filler and the matrix.

A similar trend can be observed in the case of the storage modulus where, however, for the lower level of filler loading the nature of the functionalization has a different effect on the property of the composite. The plasma functionalization with oxygen does not alter the storage modulus with respect to the value of the neat resin as a remark of the fact that there is a loss in surface hydrophilicity during composite preparation and curing, with a possible modified final interaction between the filler and the matrix. On the other hand, with a He/O_2_ plasma the effect is completely different and the final storage modulus is higher than that containing pristine graphene thanks to higher interfacial adhesion with the resin promoted by the more thermal stability of the resulted functionalization. In this case, however, the storage modulus is lower at higher concentrations probably due to a balancing of two opposite effects previously postulated in other studies [61,62,63,64,65]: the molecular confinement of the stiff filler and influence on the curing process by the polar groups on the surface of the functionalized graphene. The first determines a restrained mobility of the polymer chains due to the stronger interaction with the filler; this, in turn, implies the necessity of higher thermal energy to activate the cooperative motions related to the glass transition phenomenon. The opposite effect is due to the presence of oxygen rich functionalities on the filler surface that can alter the curing process of the resin. Specifically carboxyl and carbonyl functional group can react with the amine terminated groups of the hardener [66], at the beginning of the curing process, i.e., before the functionalization is partly lost due to high temperature treatment. This last effect reduces the crosslinking density of the final composite, which is reflected in a lower final storage modulus and also lower T_g_. Up to a certain level of filler loading, molecular confinement seems to be predominant but above a threshold value the filler could start to negatively interfere with the curing process of the epoxy resin [67].

**Figure 12:**
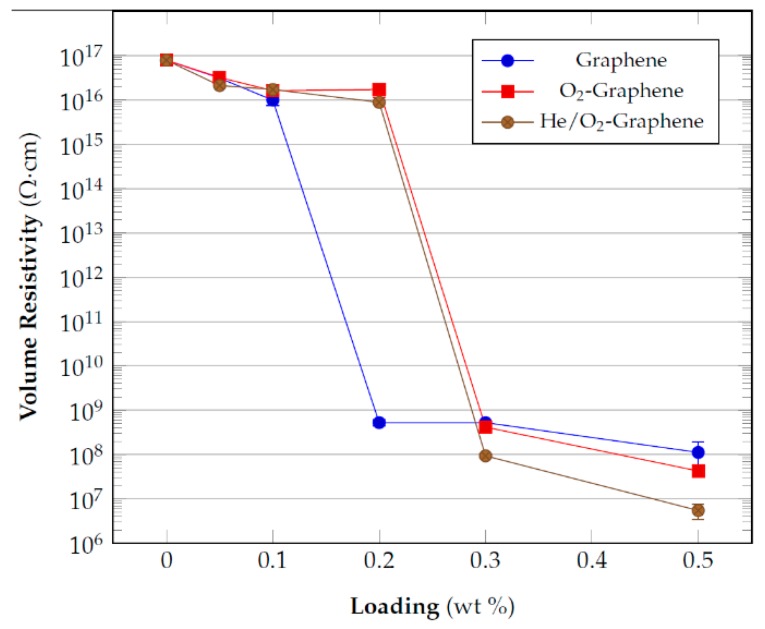
Electrical resistivity of graphene composites for pristine and functionalized filler as function of their wt% content into the epoxy matrix.

The electrical resistivity measurements (Figure 12) for the He/O_2_ plasma treated graphene samples in comparison to the epoxy composite containing the pristine and O_2_ filler have shown that the plasma treatment provide the same percolation threshold irrespective of the type of gas employed.

In this case, even if there is a loss of functionality for the oxygen plasma treated graphene, the results can be justified by the fact that the functionalization alters the structure of the material and generates defect sites on the electron π conjugation along the graphene surface which are still present whether or not the functionality is partially lost; these defects negatively affect its intrinsic conductivity.

For this reason, the plasma treatment determines a higher percolation threshold for functionalized graphene than the pristine one. A slight improvement is obtained in terms of an order of magnitude of reduction for the He/O_2_ treated samples possibly because of the better dispersion achieved in the final composites due to the retained higher functionalization.

It shall also be underlined that it is possible that optimal loading level of graphene could be achieved at concentrations intermediate between 0.05% and 0.5% in order to obtain electrical percolation along with improvement of flexural properties at the same time.

## 4. Conclusions

A screening activity has been carried out with the aim to evaluate the possible application of vacuum plasma functionalization for the production of epoxy composites with improved properties. The main aim of this work has been to assess if and how much the fillers functionalization can prevent/hinder the filler sedimentation and agglomeration during both the masterbatches storage and resin curing; moreover, also the effect of the higher compatibility between functionalized filler and resin on the mechanical and electrical resistivity of the composites has been investigated. Optimal parameters for the plasma treatment were defined after a series of tests in which different plasma parameters were changed in order to highlight their effect. Consistent results can be obtained by operating under conditions that increase the plasma density in order to provide more reactive species on the surface of the material (higher gas pressures and lower distance from the plasma source). It has been demonstrated that the functionalization is able to hinder the sedimentation process during both masterbatches storage and curing thanks to the improved compatibility with the matrix. However, a critical issue is the effect of the thermal treatment of the material during the filler dispersion and the curing process, which is capable to remove the functionalization obtained by plasma treatment. The effect of the thermal treatment on the functionalization stability depends on the heating conditions and the gas employed during the plasma process. He/O_2_ mixtures are capable to provide a more thermally stable functionalization while oxygen has the opposite effect. This can be observed in the results of the mechanical and electrical properties for the different graphitic composites which showed no improvements after the oxygen plasma treatment of the filler, while consistent results have been obtained with graphene using a He/O_2_ mixture (5:1 volumetric ratio).

The electrical resistivity measurements have shown that the plasma functionalization increases the percolation threshold for graphene composites. This result is reasonable and could be expected as a consequence of the higher disorder induced in the material structure by the acquired functionality.

## Figures and Tables

**Table 1:** Summary of the variables taken into consideration the study of the effect of plasma treatment on graphite functionalization.

Variable	Level/Condition of Treatment
Gas for plasma atmosphere, Gas	O_2_, Ar, Air, N_2_
Plasma power, MW [W]	80, 100, 200
Residence time, RT [min]	1, 2, 5, 10, 30
Chamber pressure, P [mbar]	0.01, 0.06
Plasma source-sample distance, d [cm]	3, 6
Amount of treated material, m [g]	0.25, 1, 2, 3, 4, 5

**Table 2:** Effect of microwave (MW) plasma operative parameters on graphite hydrophilicity. Best conditions for the functionalization are reported in bold as comparison with the other combinations of variables (CW = complete wetting, <30° values refer the formation of a drop on the graphite surface but a precise measure is not accurate to be reliable).

m [g]	MW [W]	Gas	RT [min]	P [mbar]	d [cm]	Contact Angle [°]							
						12 h	4 days	7 days	14 days	30 days	45 days	60 days	75 days
0.25	80	Air	10	0.06	6	60.4 ± 1.4	-	-	-	-	-	-	-
		O_2_				56.3 ± 1.6	-	-	-	-	-	-	-
0.25	80	O_2_	5	0.06	6	55.9 ± 2.1	-	-	-	-	-	-	-
					3	CW	CW	CW	CW	<30	<30	<30	<30
0.25	80	O_2_	1	0.06	3	CW	CW	CW	CW	<30	<30	<30	55.5 ± 2.3
			2			CW	CW	CW	CW	CW	<30	<30	<30
			5			CW	CW	CW	CW	<30	<30	<30	<30
			10			CW	CW	CW	CW	<30	<30	<30	<30
0.25	80	O_2_	2	0.01	3	<30	<30	<30	<30	-	-	-	-
				0.06		CW	CW	CW	CW	CW	<30	<30	<30
0.25	80	O_2_	2	0.06	3	CW	CW	CW	CW	CW	<30	<30	<30
		N_2_				<30	<30	<30	<30	<30	<30	<30	51.3 ± 2.1
		Air				<30	<30	<30	<30	<30	<30	<30	55.2 ± 1.4
		Ar				<30	<30	<30	<30	<30	<30	<30	46.4 ± 0.8
0.25	80	O_2_	2	0.06	3	CW	CW	CW	CW	CW	<30	<30	<30
	100					CW	CW	CW	CW	CW	<30	<30	<30
	200					CW	CW	CW	CW	CW	<30	<30	<30
0.25	80	O_2_	2	0.06	3	CW	CW	CW	CW	CW	<30	<30	<30
1						<30	<30	<30	<30	-	<30	38.4 ± 1.9	40.3 ± 2.0
2						<30	<30	<30	<30	-	51.8 ± 3.2	59.1 ± 1.5	61.4 ± 1.5
3						<30	<30	<30	<30	-	43.2 ± 1.8	50.1 ± 1.8	53.4 ± 2.0
4						<30	<30	<30	<30	-	57.4 ± 1.8	66.3 ± 2.0	67.1 ± 1.4
5						<30	<30	<30	<30	-	61.5 ± 2.2	67.2 ± 0.5	71.4 ± 1.1
0.25	80	O_2_	2	0.01	3	<30	<30	<30	<30	-	-	-	-
	100					56.0 ± 2.3	-	-	-	-	-	-	-
	200					50.0 ± 3.1	-	-	-	-	-	-	-

**Table 3:** Effect of the different microwave (MW) plasma operative parameters on the hydrophilicity of graphene (CW = complete wetting of the samples surface).

m [g]	MW [W]	Gas	RT [min]	P [mbar]	d [cm]	Contact Angle [°]							
						12 h	4 days	7 days	14 days	30 days	45 days	60 days	75 days
0.05	80	O_2_	2	0.06	3	CW	CW	CW	CW	CW	-	CW	CW
	200					CW	CW	CW	CW	CW	-	62.4 ± 3.4	64.6 ± 1.3
	80	O_2_	2	0.06	3	CW	CW	CW	CW	CW	-	CW	CW
		Air				CW	CW	CW	CW	CW	-	45.4 ± 2.2	44.1 ± 1.5
	80	O_2_	2	0.01	3	CW	CW	CW	CW	CW	-	CW	CW
				0.06		CW	CW	CW	CW	CW	-	CW	CW
	80	O_2_	1	0.06	3	CW	CW	CW	CW	CW	-	CW	65.2 ± 1.2
			2			CW	CW	CW	CW	CW	-	CW	CW

**Table 4:** Microwave (MW) plasma treatment parameters and results of water contact angle for graphene treated with gas mixtures (CW = complete wetting of the samples surface).

m [g]	MW [W]	Gas	RT [min]	P [mbar]	d [cm]	Contact Angle [°]
						12 h
0.25	80	He	2	0.06	3	CW
		Ar				
		He/O_2_ (10:1)				
		He/O_2_ (5:1)				
		He/O_2_ (2:1)				
		Ar/O_2_ (10:1)				

**Table 5:** Contact angles and plasma parameters for thermal treated graphene samples (CW = complete wetting of the samples surface).

Gas	Plasma Parameters				Treatment	Contact Angle [°]
	Microwave Power [W]	Time [min]	Pressure [mbar]	Distance [cm]		
None	80	2	0.06	3	12 h @ 120 °C	109 ± 2
O_2_	80	2	0.06	3	Not treated	CW
					1 h @ 80 °C	58 ± 1
					12 h @ 80 °C	98 ± 1
He	80	2	0.06	3	Not treated	CW
					1 h @ 80 °C	56 ± 2
					12 h @ 80 °C	51 ± 1
					30′ @ 160 °C	47 ± 1
					2 h @ 160 °C	49 ± 1
Ar	80	2	0.06	3	Not treated	CW
					1 h @ 80 °C	49 ± 3
					12 h @ 80 °C	56 ± 1
					30′ @ 160 °C	44 ± 1
					2 h @ 160 °C	61 ± 2
He/O_2_ (10:1)	80	2	0.06	3	Not treated	CW
					1 h @ 120 °C	CW
					12 h @ 120 °C	81 ± 3
He/O_2_ (5:1)	80	2	0.06	3	Not treated	CW
					1 h @ 120 °C	CW
					12 h @ 120 °C	67 ± 2
He/O_2_ (2:1)	80	2	0.06	3	Not treated	CW
					1 h @ 120 °C	CW
					12 h @ 120 °C	93 ± 2

**Table 6:** Flexural toughness values obtained from stress-strain curves.

Sample	Toughness [J/m^3^ *10^4^]
neat	213
0.05%	114
0.05% O_2_	80
0.05% O_2_ RT	73
0.05% He–O_2_	121
0.5%	105
0.5% He–O_2_	109

**Table 7:** Effect of plasma functionalization on the storage modulus and T_g_ of graphene filled epoxy composites.

Sample	Storage Modulus [MPa]	T_g_ from Tan δ [°C]
Neat resin	1624	123
0.05% Graphene	1831	143
0.05% O_2_-Graphene	1617	140
0.05% He/O_2_-Graphene	1927	144
0.5% Graphene	1854	136
0.5% He/O_2_-Graphene	1807	135
